# IRMPD Spectroscopy of Homo- and Heterochiral Asparagine Proton-Bound Dimers in the Gas Phase

**DOI:** 10.1021/acs.jpca.1c05667

**Published:** 2021-08-24

**Authors:** Åke Andersson, Mathias Poline, Kas J. Houthuijs, Rianne E. van Outersterp, Giel Berden, Jos Oomens, Vitali Zhaunerchyk

**Affiliations:** † Department of Physics, University of Gothenburg, 41296 Gothenburg, Sweden; ‡ Department of Physics, Stockholm University, 10691 Stockholm, Sweden; § FELIX Laboratory, Institute for Molecules and Materials, Radboud University, Toernooiveld 7, 6525 ED Nijmegen, The Netherlands

## Abstract

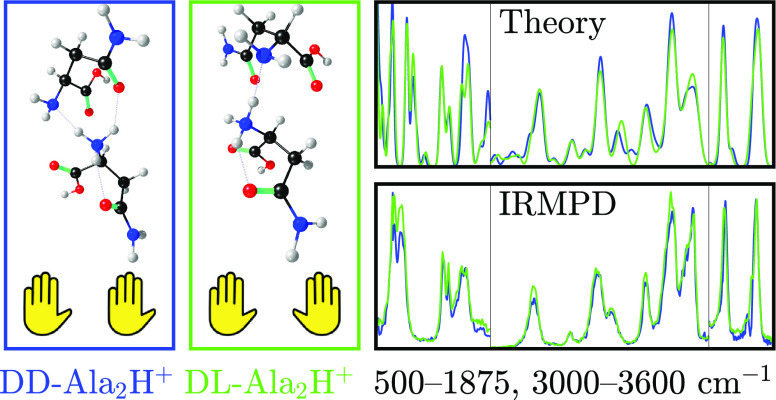

We investigate gas-phase structures of homo- and heterochiral asparagine proton-bound dimers with infrared multiphoton dissociation (IRMPD) spectroscopy and quantum-chemical calculations. Their IRMPD spectra are recorded at room temperature in the range of 500–1875 and 3000–3600 cm^–1^. Both varieties of asparagine dimers are found to be charge-solvated based on their IRMPD spectra. The location of the principal intramolecular H-bond is discussed in light of harmonic frequency analyses using the B3LYP functional with GD3BJ empirical dispersion. Contrary to theoretical analyses, the two spectra are very similar.

## 1 Introduction

Investigation of amino acids (AAs) and their oligomers are of interest because of their fundamental role in biology. Protonation of an AA dramatically alters its structure by changing which inter- and intramolecular noncovalent interactions (NCI) to the environment are preferred.^1−7^ Gas-phase proton-bound AA dimers, meaning pairs of AAs with one extra H^+^, have gained significant interest, both from experimental and theoretical perspectives^8−16^ because they contain the complexity of inter- and intramolecular NCI, despite their small size. This makes them useful model systems for the NCI in proteins.

Investigations of these dimers often use action spectroscopy techniques such as infrared multiple photon disassociation (IRMPD) spectroscopy.^1,2,8,10,12,14,17^ The action spectrum of IRMPD approximates the absorption spectrum in the infrared (IR) range.^18^ IRMPD spectroscopy combined with theoretically predicted IR spectra can therefore be used to deduce the three-dimensional (3D) structure of molecular systems.

Proton-bound dimers can assume many conformers, commonly classified into two categories: charge-solvated (CS) or salt-bridge (SB) structures, the latter defined by one monomer being zwitterionic. In the gas phase, dimers are likely to be CS in their ground state if the constituent monomers have a relatively low proton affinity (PA)^11,19^ or can stabilize through interaction with the side chain.^11,20^ Asparagine (Asn) is on the threshold, with a PA that is within calculation error of the PA of proline and threonine, which are SB^10^ and CS,^11^ respectively. The structure of the protonated asparagine dimer has not been investigated before, to the best of our knowledge.

All proteinogenic amino acids except glycine are chiral and naturally occur in the l-configuration. Chiral-specific interactions determine many processes occurring in organisms including odor perception and efficiency of medicines. Gas-phase proton-bound AA dimers with moieties in different chiral configurations are attractive model systems for studies of chiral-specific interactions at the most fundamental level. IRMPD spectroscopy has successfully been used to discern chiral differences in gas-phase molecules.^17,21−23^

We have previously investigated homo- and heterochiral proton-bound dimers theoretically.^15^ Our findings suggest that for chiral differences to appear in the mid-IR spectra, there must be intermolecular interactions with the side chain, which is the case for asparagine because the O- and N-atoms on its side chain enable H-bonds.

In this paper, we report IRMPD spectra of proton-bound asparagine dimers in two chiral configurations: homo- and heterochiral. We infer that the two differ in which sets of intramolecular interactions are possible. There exist vibrational modes whose stiffness comes in part from such interactions; thus, one may expect frequencies to shift relative to the monomer. To enhance the chance to unravel IR diastereomer-specific features, both diastereomers are measured simultaneously. This prevents features from being obscured by fluctuations in laser beam power. Simultaneous measurement is achieved by mass labeling the heterochiral dimer.

## 2 Methods

### 2.1 Experiment

The experimental work was done at the Free-Electron Laser for Infrared eXperiments (FELIX) facility at Radboud University in Nijmegen, the Netherlands. Proton-bound dimers of asparagine were studied in two chiral configurations: the homochiral dd-Asn_2_H^+^ and the heterochiral dl-Asn_2_H^+^. For the sake of labeling, the l-Asn monomer was an isotopologue; both N-atoms were ^15^N. As a consequence, the two dimer diastereomers were completely separable in mass. This allowed for simultaneous measurement of the two diastereomers’ IR spectra, which eliminates the risk of observing false differences caused by experimental conditions, such as fluctuating laser power.

An asparagine solution was prepared as a mixture of d-Asn and l-Asn dissolved in a 49:49:2 mixture of water, methanol, and formic acid at a concentration of 1 μM. The dimer ions were generated via electrospray ionization and stored in a 3D quadrupole ion trap mass spectrometer (MS, Bruker amaZon speed ETD).^24^ The heterochiral dimer, dl-Asn_2_H^+^ (*m* = 267 Da), and one kind of homochiral dimer, dd-Asn_2_H^+^ (*m* = 265 Da) or ll-Asn_2_H^+^ (*m* = 269 Da), were simultaneously isolated. The trapped ions were irradiated with a single pulse from the infrared FEL (*f*_FEL_ = 500–1875 cm^–1^, *E*_pulse_ = 30–160 mJ). In the MS, the abundances of dimers and their IR-induced monomer fragments were measured after the irradiation.

The experiment described above was then repeated with the following two changes: FELIX was substituted for a tabletop LaserVision OPO laser, which was used in the range of 3000–3600 cm^–1^, with a pulse energy on the order of 10 mJ and a repetition rate of 10 Hz. The MS was a Fourier transform ion cyclotron resonance (FTICR) mass spectrometer.^25^

Given the abundances *I*_DD_, *I*_DL_, *I*_D_, and *I*_L_ of the two dimers and the two monomers, the IRMPD intensities of dd-Asn_2_H^+^ and dl-Asn_2_H^+^ are calculated as
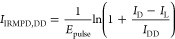
1
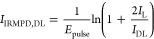
2These equations are derived from the fact that dd-Asn_2_H^+^ always fragments into d-AsnH^+^, and dl-Asn_2_H^+^ fragments into d-AsnH^+^ and l-AsnH^+^ with equal probability. Dividing by pulse energy corrects for its frequency dependence, assuming a linear dependence of IRMPD fragmentation yield as a function of laser pulse energy.^26^ When the homochiral dimer is instead ll-Asn_2_H^+^, every instance of d and l must be exchanged for the other.

### 2.2 Calculations

A conformational search was performed using several molecular dynamics (MD) simulations with different initial geometries to cover a large conformation space. The MD calculations were carried out in the microcanonical ensemble employing the density functional-based tight binding method^27^ as implemented in the DFTB+ software package.^28^ The initial velocities were chosen to correspond to a Maxwell–Boltzmann distribution at 298 K, and a velocity Verlet algorithm with a time step of 1 fs was implemented. The added mass of ^15^N was considered in l-AsnH^+^ moieties.

The most stable structures obtained with the MD simulations were optimized with density functional theory (DFT) using three separate functionals: B3LYP with GD3BJ dispersion, ωB97XD, and M06-2X, all together with the 6-311++G** basis set. The B3LYP-GD3BJ functional was also combined with the N07D^29^ and aug-cc-pVDZ basis set. For structures optimized with the 6-311++G** basis set, single-point energies were calculated with the G4MP2 method,^30,31^ which is known to be accurate for this task.^32^ To obtain the corresponding Gibbs energies, G4MP2 calculations were combined with the vibrational analyses performed at the same level as optimization. Conformers with and without the l-Asn moiety labeled were both considered, and their energies were found to differ only by a negligible amount. The harmonic frequency analyses were performed using the same five methods used for optimization. All of these calculations were carried out with the Gaussian 16 program.^33^

Before comparing the frequency analyses of conformers with an experimental spectrum, the prediction is transformed in two ways: broadening and scaling. First, the discrete IR spectrum of every conformer is broadened into a continuous one by transforming each vibrational mode with frequency *f* and intensity *I* into a Gaussian function with mean μ = *f*, and integral *I*. The width is chosen to match the experimental spectra, σ = 0.01*f* (full width at half-maximum (FWHM) = 0.0235*f*) in the FEL range, and σ = 0.003*f* (FWHM = 0.00471*f*) in the OPO range. Second, frequencies are scaled by constants, typically 0.98 (0.95) when the frequency is below (above) 2000 cm^–1^. These scaling factors improve accuracy by partially accounting for anharmonicity in vibrational modes.^20,34^

When quantifying the accuracy of predicted frequencies, we use two measures: root-mean-square error (RMSE) and weighted linear correlation distance. The RMSE is computed as
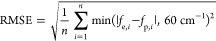
3where *f*_e,*i*_ is the *i*th experimental peak position and *f*_p,*i*_ is the corresponding assigned predicted peak position, or 0 if none exists. The constant 60 cm^–1^ is arbitrarily taken to be about 3 times the typical deviation. The weighted linear correlation distance  of the entire spectrum is summarized from  on three intervals. It is intuitively understood as the answer to the question “How close (in the square-norm sense) is the predicted spectrum to the experimental, if we allow linear scaling of intensity on each interval?” and is closely related to the cosine similarity score *S*.^35^ Explicitly
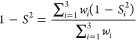
4
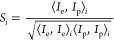
5
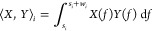
6where *I*_e_(*f*) is the experimental and *I*_p_(*f*) is the predicted intensity at frequency *f*. (*s*_1_, *s*_1_ + *w*_1_) = (500, 900) cm^–1^, (*s*_2_, *s*_2_ + *w*_2_) = (1050, 1850) cm^–1^, and (*s*_3_, *s*_3_ + *w*_3_) = (3350, 3600) cm^–1^ are the three intervals of interest. Subdivision into three intervals is motivated by the variation of intensity. Without subdivision, intense features in the range of 1500–1850 cm^–1^ would dominate the analysis.

## 3 Results and Discussion

### 3.1 IRMPD Experiment

**Figure 1:**
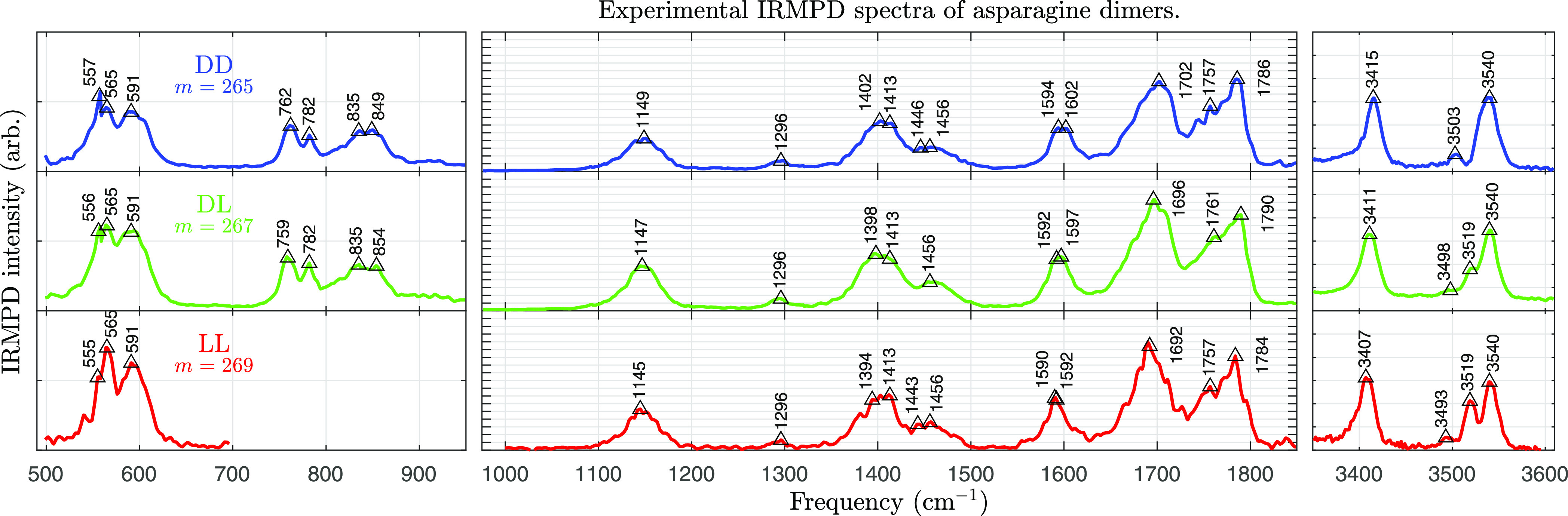
Experimental IRMPD spectra of (top) dd-Asn_2_H^+^, (middle) dl-Asn_2_H^+^, and (bottom) ll-Asn_2_H^+^. The IRMPD intensity is averaged over several scans. Very broad spectral features attributable to bands involving a shared proton are found in the frequency range of 3000–3300 cm^–1^ and presented in Figure S3 in the Supporting Information.

Figure 1 shows the obtained experimental IRMPD spectra of dd-Asn_2_H^+^, dl-Asn_2_H^+^, and ll-Asn_2_H^+^. Spectra from multiple scans have been averaged.

There are differences in peak positions between the spectra in Figure 1. Many peaks become red-shifted as the mass of the dimer increases. This is exactly what should be expected for vibrational modes that involve N: the isotopically labeled N-atoms (^15^N) increase the effective mass of the mode, causing the frequency to decrease. The shift therefore indicates whether a peak corresponds to a vibrational mode that displaces N-atoms.

### 3.2 Structure and Energy

Structures are classified as salt-bridged (SB) if the unprotonated moiety is zwitterionic, and charge-solvated (CS) otherwise. CS structures are further classified as type A or B when the dominant intermolecular interaction is NH_3_^+^···NH_2_ or NH_3_^+^···COOH, respectively.^14,20,36^ Similarly, SB is classified as type Z when it is NH_3_^+^···COO^–^. We give conformers names based on chirality of the monomers (dd-, dl, or ll), the structure type (A, B, or Z), and electronic energy rank among conformers of the same chirality and structure type. We chose electronic (rather than Gibbs) energy for this purpose because it can be calculated with great accuracy using G4MP2//B3LYP-GD3BJ/6-311++G**.

**Figure 2:**
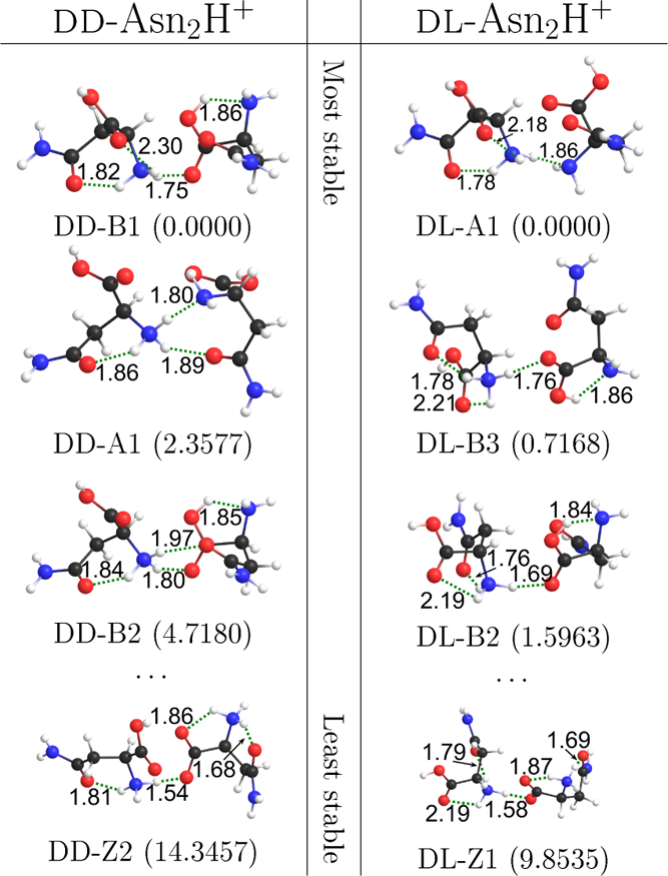
Three conformers of dd-Asn_2_H^+^ and dl-Asn_2_H^+^, according to G4MP2//B3LYP-GD3BJ/6-311++G**. The most stable conformer of type Z is also included. Calculated relative Gibbs energies are given in kJ mol^–1^. Weak inter- and intramolecular interactions are shown as dashed lines annotated with their length in angstrom.

The three lowest Gibbs energy conformers of each dimer according to G4MP2//B3LYP-GD3BJ/6-311++G** are shown in Figure 2. They are all CS, so we also include the most stable SB conformer for comparison. The SB structures all have too high energy to be populated, regardless of what method is used. Therefore, it is likely that asparagine dimers are CS.

The Gibbs energy ranking of conformers is significantly dependent on the choice of the DFT method for calculating frequency. For instance, at 300 K, dd-B1 is predicted to be the most abundant by B3LYP-GD3BJ, while its relative abundance is less than 5% when using M06-2X, and the relative abundance of type B structures changes as a result. For a full comparison, see Figure S4 in the Supporting Information.

For the homochiral dimer, conformers differentiate more in energy. To minimize the risk of missing a homochiral conformer, we analyzed the intermolecular interactions of stable heterochiral conformers and created corresponding homochiral conformers. However, after optimization, these only yielded high energy or already known conformers.

### 3.3 Spectra Assignment

**Figure 3:**
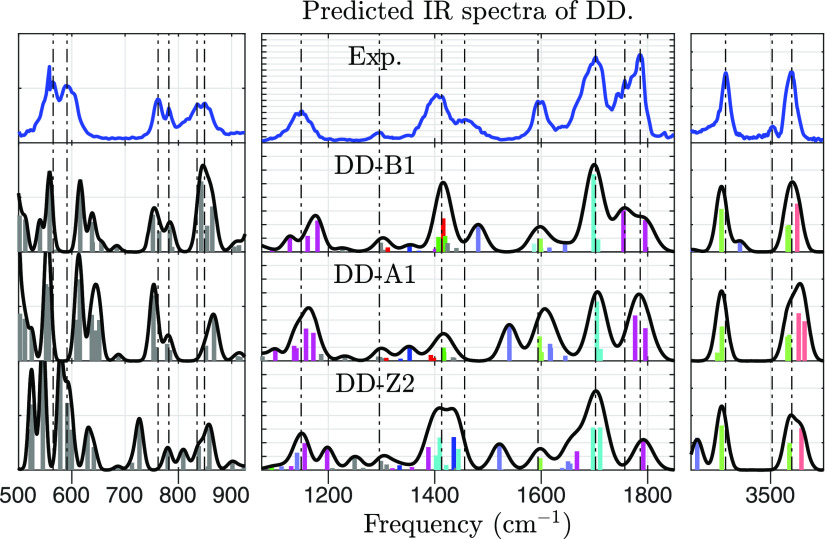
Predicted spectra of abundant dd-Asn_2_H^+^ conformers compared with the experiment. B3LYP-GD3BJ/N07D is used for calculating IR frequencies, which are then scaled and broadened. The color of an IR band tells the locus of the corresponding vibrational mode. Red, green, and blue components imply the movement of OH, side-chain NH_2_, and α-NH_2_, respectively. A brighter color implies that no C-atoms move.

**Figure 4:**
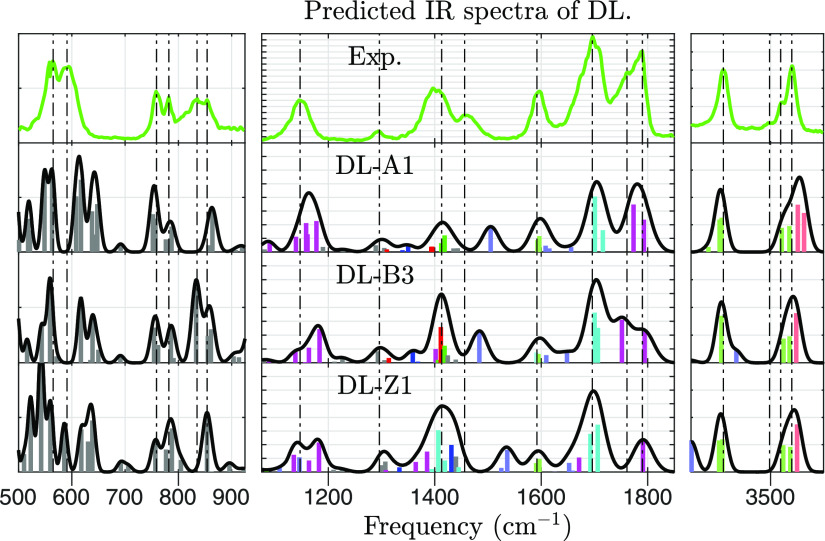
This figure is to dl-Asn_2_H^+^ as Figure 3 is to dd-Asn_2_H^+^. The ^15^N-labeled l-AsnH^+^ moiety is assumed to be protonated or unprotonated with equal probability.

A comparison between the experimental IRMPD spectra from Figure 1 and theoretically predicted IR frequencies is made in Figures 3 and 4. What follows is an assignment of vibrational modes to the observed features.

- Around 580 cm^–1^, a broad twin peak is seen, but more than two peaks are predicted regardless of conformer type, all related to delocalized vibrational modes spanning entire moieties.
- Around 770 cm^–1^, another twin peak is observed. It is well predicted by intense modes in CS structures (A and B). SB structures (Z), on the other hand, are predicted to have a strong peak at 720 cm^–1^ for the homochiral dimer, contrary to the experiment. From this, we infer that type Z is likely not populated, which was also predicted from theoretical calculations on abundances.
- Around 845 cm^–1^, there is yet another twin peak. It aligns best with predictions from type B conformers, but also reasonably well with the alternatives. The matching vibrational modes mostly involve NH_2_ wagging, and its recoil spread throughout the moiety.
- At 1149 cm^–1^, a broad peak matches the predicted frequency of (magenta) amino acid vibrational modes, in which the COOH and α-NH_*x*_ functional groups move. The peak is slightly shifted in Figure 4 by the isotopologue effect, which is consistent with the fact that N-atoms participate in the mode.
- At 1296 cm^–1^, a small peak matches the predicted frequency of (gray) CH_2_-wagging. As expected, no isotopologue shift is observed.
- Around 1400 cm^–1^, a broad and wavy peak is seen, suggesting the presence of overlapping IR bands. Indeed, multiple vibrational modes are predicted around this frequency. Most are (green) H_2_N–C=O or (red) HO–C=O bending. The summed intensity is greater for conformers of type B than type A, and this favors type B when comparing with the experiment.
- At 1456 cm^–1^, the experimental spectrum contains a peak that is not matched by theory. We believe that it corresponds to (bright blue) NH_3_ umbrella bending, whose frequency is predicted to be ≈1500 cm^–1^. This mode is likely red-shifted compared to a prediction within the harmonic approximation as the H-atoms participate in intramolecular interactions, which gives the vibration an anharmonic nature. In support of this, no peak is seen at 1500 cm^–1^. Due to this anharmonicity, we cannot infer on which type is favored from this mode. No isotopologue shift is observed, which can be explained by the fact that the N-atom is quite stationary in NH_3_ umbrella bending.
- At 1598 cm^–1^, a peak with a slight twin structure is seen. This matches the predicted frequency of NH_*x*_ scissoring of the (bright blue) α-NH_*x*_ and (bright green) side-chain NH_2_ functional groups. In type B conformers, there is also a (bright cyan) coupled scissoring between the two NH_*x*_ groups. Isotopologue shift is observed as expected.
- At 1702 cm^–1^, a strong peak matches the predicted frequency of (cyan) side-chain C=O stretching, which is combined with the side-chain NH_2_ group, and to a lesser extent, the α-NH_*x*_ group. Indeed, this peak has a significant isotopologue shift.
- At 1786 cm^–1^, another strong peak is seen, with a shoulder bump at 1757 cm^–1^. These match predictions for (magenta) COOH stretching, which is coupled with the α-NH_*x*_ group. The predictions differ between structure types. In type A, both COOH-groups are free, and their stretching frequencies are both near 1786 cm^–1^. However, in type B, one COOH participates in the principal intermolecular interaction, which shifts its frequency to 1750 cm^–1^. In the experiment, we observe this as a shoulder, implying the presence of type B. However, the relative prominence of the peaks is closer to the predicted spectra of type A. Estimating a relative abundance of conformers A and B from the intensities of these bands is unwarranted because the intensities of IRMPD peaks may differ from those of linear (single-photon) IR spectra,^37,38^ and computed harmonic intensities may be off.
- In the range of 3000–3300 cm^–1^ (Figure S3 in the Supporting Information), there is a large discrepancy between the theory and experiment. All levels of theory predict XH stretching modes, and in many of these, the H-atom participates in an inter- or intramolecular interaction. However, the experimental spectra show only one diffuse feature spread over the whole range. Similar results are reported for other AA dimers or clusters of proline, serine, and threonine.^11,22^
- At 3415 cm^–1^, a peak matches the predicted frequency of (bright green) symmetric side-chain NH_2_ stretching. As expected, there is an isotopologue shift.
- At 3503 cm^–1^, there is a small peak that matches no predicted frequency well. We believe that it corresponds to the (bright blue) α-NH_2_ asymmetric stretch, whose frequency is predicted to be 3440 cm^–1^. No peak is observed at this frequency, so it is reasonable to assume it is shifted. The α-NH_2_ is only free to stretch in conformers of type B, in all of which it has an intramolecular interaction with the H from the COOH group. There is an isotopologue shift.
- At 3540 cm^–1^, a peak matches the predicted frequency of both (bright green) asymmetric side-chain NH_2_ and (bright red) OH stretching. These modes are not resolved for the unlabeled dimer. Contrapositively, when the dimer is labeled, the NH_2_ stretch appears at 3519 cm^–1^, clearly distinct from the OH stretch.

In summary, every observed peak is explained by harmonic theory on the level of B3LYP/N07D. Only two observed peaks appear at different frequencies than predicted. These were the α-NH_3_ umbrella bend and α-NH_2_ antisymmetric stretch modes. Both of these functional groups participate in H-bonds, which are known to be highly anharmonic. Based on the appearance of the α-NH_2_ antisymmetric stretch mode, we infer that a conformer of type B is largely populated. On the other hand, based on the relative prominence of the peak at 1790 cm^–1^, we suspect that a conformer of type A is also populated.

### 3.4 Method Errors

**Figure 5:**
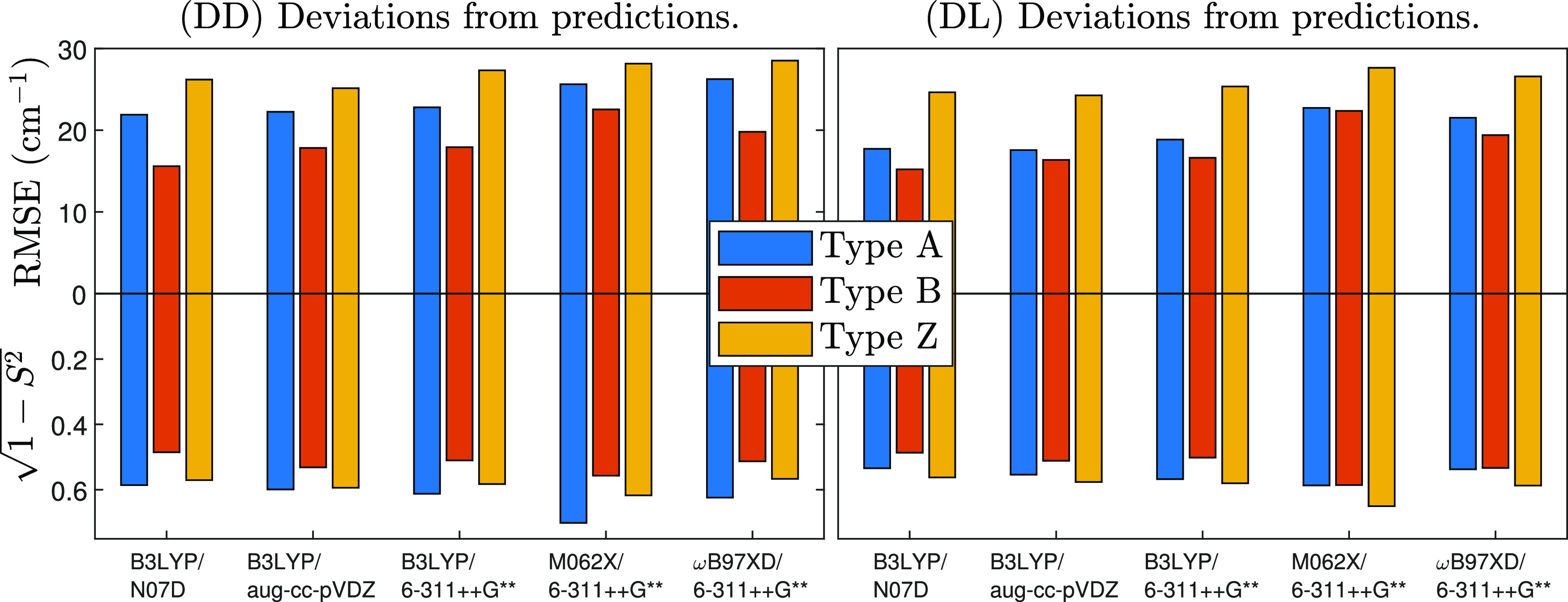
Deviations between experimental and predicted spectra using dual scaling. The most stable conformer of each structure type is used. The top half shows the root-mean-square error of predictions. Whenever a method fails to predict an observed peak, a penalty is given, equivalent to a 60 cm^–1^ error. The bottom half shows the linear correlation distance  measure of spectra agreement. Regardless of the computational method or error measure, the type B conformer fits the experiment best.

With the purpose of eliminating dependence on a specific method, vibrational frequency analyses were repeated, employing a total of five methods: B3LYP-GD3BJ with the N07D, aug-cc-pVDZ, and 6-311++G** basis sets; and additionally ωB97XD and M06-2X with the 6-311++G**basis set. Figure 5 compares the errors of these five methods on the most stable conformer of each type, using the two quantitative measures from eqs 3 to 6. Frequencies are scaled with two fitted factors, above and below 2000 cm^–1^. The RMSE measure uses 18 observed and assigned peaks.

The results show that predictions from type B structures match the experiment better, regardless of the computational method or error measure. This reinforces the earlier result that a conformer of type B is populated because the  measure is objective in the sense that it is independent of peak assignment.

Of all methods, B3LYP-GD3BJ/N07D has the lowest RMSE, 16 cm^–1^. This is a slightly optimistic estimate of the accuracy of the method because we fit two scaling factors to the spectrum of one molecule. B3LYP is indeed accurate for frequencies,^34^ especially so with N07D, as inferred from a study of aminophenol isomers.^39^

### 3.5 Chiral Effects

From the spectra in Figure 1, some possible chiral effects can be inferred. Near 1445 cm^–1^, the homochiral dimer spectra have a slight peak, while the heterochiral dimer spectrum has a valley. Around 1760 and 1790 cm^–1^ the heterochiral dimer peak position deviates from the average of the two homochiral dimers. All three peaks correspond to vibrations of functional groups that participate in the principal H-bond, lending credence to the hypothesis that these differences are caused by chiral-specific intermolecular interactions.

**Figure 6:**
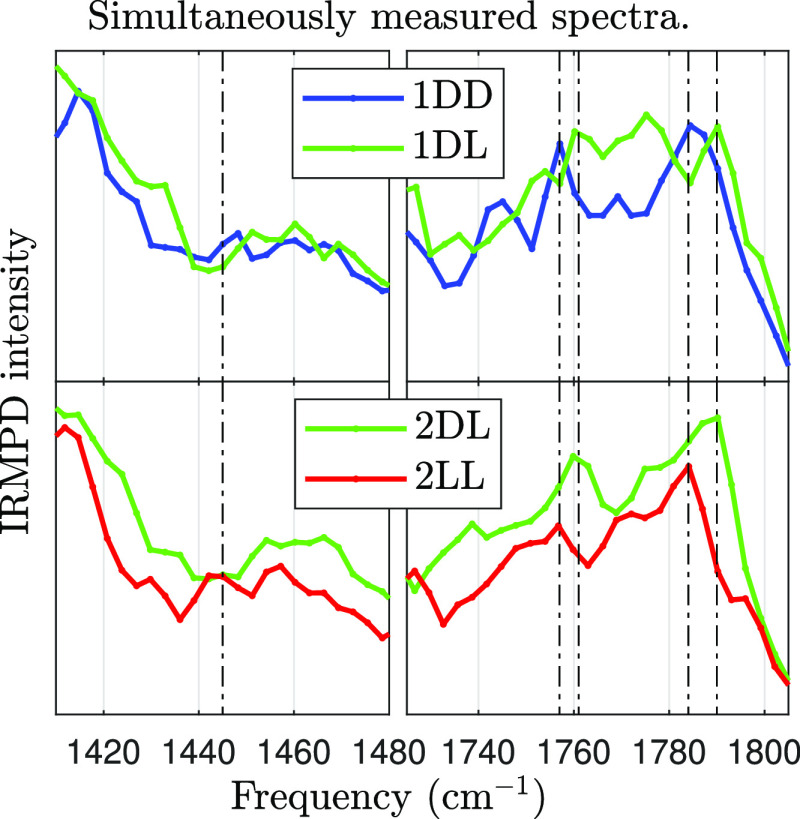
Simultaneously measured spectra of the homo- and heterochiral dimer. Each row represents a single experimental scan. Vertical lines trace possible chiral effects.

Because these effects are very small, it is imperative to look at simultaneous measurements of the two diastereomers. Figure 6 shows two scans of the regions where chiral effects are suspected, in which the IRMPD intensities of the homo- and heterochiral dimer are measured simultaneously and derived according to eq 2. Both scans contain the chiral effects seen in the unified spectra. At 1445 cm^–1^, only the homochiral dimer has a small peak. Near 1760 and 1790 cm^–1^, a peak is red-shifted for the homochiral dimer.

To quantify the magnitude of any chiral effects, we calculate the Pearson’s ρ correlation coefficient between the four spectra in Figure 6 in the ranges 1410–1480 and 1725–1795 cm^–1^. The correlation is slightly stronger between similar diastereomers from different scans (ρ(1dd,2ll) = 90%, ρ(1dl,2dl) = 90%) compared to different diastereomers from the same scans (ρ(1dd,1dl) = 86%, ρ(2dl,2ll) = 83%). This remains true if the linear correlation coefficient is used instead of Pearson’s.

Harmonic frequency calculations suggest that the frequency of the NH_3_ umbrella bending mode is conformer-dependent, and thus a possible indicator of diastereomer type (homo- or heterochiral). This frequency is 1477–1503 cm^–1^ for most of the abundant conformers, with the noticeable exception of dd-A1, for which it is 1540 cm^–1^. The higher frequency of NH_3_ umbrella bending in dd-A1 is intuitively explained by the fact that this group participates in three strong H-bonds. As it is argued in the previous section, we suspect that the structure of the homochiral dimer in our experiment is predominantly of type B and therefore does not show the large frequency shift associated with dd-A1.

Fortunately, dd-A1 has by far the lowest electronic energy among conformers of dd-Asn_2_H^+^, meaning it can be forced by thermalization in a cryogenic environment. According to Gibbs energies from calculations with B3LYP-GD3BJ/6-311++G**, at temperatures lower than 100 K both dd-A1 and dl-A1 are populated more than 95% in their respective diastereomers. The frequency shift of the NH_3_ umbrella bending mode is predicted to be 35 cm^–1^ and should therefore be resolvable with a line width below 2%.

## 4 Conclusions

The IRMPD spectra of homo- and heterochiral proton-bound asparagine dimers were obtained in the frequency ranges of 500–1875 and 3000–3600 cm^–1^. The observed spectra are best matched by charge-solvated conformers of type B, but the spectra around 1800 cm^–1^ suggest that a conformer of type A is populated to some degree. The results are intriguing, since it is believed that CS structures are favored when the PA is low or when the protonated amino group interacts with the side chain of the unprotonated moiety. This is generally not the case in type B conformers. Theoretical Gibbs energy calculations confirm that the dimer is CS, but the total relative abundance of conformers of type A varies depending on the method.

The structure of proton-bound dimers is known to be related to the PA of the monomer, with CS (ZW) structures corresponding to low (high) PA.^11,36^ Knowing that Asn_2_H^+^ is CS helps pinpoint the critical PA. According to PA values calculated at the G3MP2 level,^40^ amino acids near the threshold and their corresponding proton-bound dimer structures are (in order of increasing PA) phenylalanine (CS), tyrosine (CS), asparagine (CS), methionine (mixed at 300 K), tryptophan (CS), and proline (SB).^10,20,41^

The relative Gibbs energies of conformers calculated with G4MP2 showed significant dependence on the choice of the optimization method because ZPE and thermal corrections depend on vibrational frequencies, which are calculated by the optimization method. In the worst case, the result varies by more than 3 *k*_B_*T* at room temperature, and not even the sign is certain. We conclude that for this purpose, at least two out of the optimization methods B3LYP-GD3BJ, ωB97XD, and M06-2X cannot be fully trusted in tandem with single-point G4MP2 calculations.

Using B3LYP-GD3BJ/N07D for geometry optimization and vibrational analyses gives great agreement with the experiment overall, but two failures are worth highlighting: first, none of the predicted frequencies in the range of 3000–3300 cm^–1^ are observed. Second, frequencies of vibrational modes of NH_*x*_ that participate in H-bonds are predicted with an error of up to 100 cm^–1^. These failures are expected because the modes are anharmonic.

Our theoretical calculations suggest that mostly dimers with limited interaction with the side chain are energetically favored at room temperature. This may explain the absence of chiral effects in the present experiment. dd-B1, dl-A1, dl-B3 are the three lowest energy conformers, though it should be noted that dd-A1 does have interaction with the side chain. This may explain the absence of chiral effects. This differs from a recent study of glutamic acid dimers^21^ in which strong interaction between the side chains was observed, resulting in clear chiral effects in the spectra. However, it could also be that the method used, IR–IR hole burning spectroscopy,^21^ is more appropriate in probing conformer-specific interactions.

Although our measurements did not reveal any prominent chiral effects, our theoretical calculations highlight the direction for future studies. According to theoretical predictions, at temperatures lower than 100 K, only one conformer is populated for both diastereomers, dd-A1 and dl-A1. These two structures have different intermolecular bonds on NH_3_, and therefore, it might be expected that vibrational bonds involving this part of the molecule will be different for the two diastereomers. Indeed, the harmonic analysis predicts a 35 cm^–1^ shift of the NH_3_ umbrella mode located around 1500 cm^–1^. While harmonic methods are inaccurate for these modes, we believe that a large predicted shift implies some experimental difference. Thus, we believe that measurements performed at cryogenic temperatures will be more prone to chiral effects in Asn proton-bound dimers. Such measurements can potentially be performed in cryogenic traps and storage rings.^42,43^

## Supporting Information Available

Experimental IRMPD spectra in the range of 500–1000 cm^–1^ (Figure S1); experimental IRMPD spectra in the range of 1000–1900 cm^–1^ (Figure S2); experimental IRMPD spectra in the range of 3000–3600 cm^–1^ (Figure S3); computed relative (top) electronic and (bottom) Gibbs energies (Figure S4); and relative electronic and Gibbs energies (Table S1) (PDF)
