# Supplementary material for: IRMPD Spectroscopy of Homo- and Heterochiral Asparagine Proton-Bound Dimers in the Gas Phase
Source: J Phys Chem A. 2021 Aug 24;125(34):7449–56. doi: 10.1021/acs.jpca.1c05667 (PMC8419839; doi:10.1021/acs.jpca.1c05667)
Supplement: Supplementary file 1 — jp1c05667_si_001.pdf [file jp1c05667_si_001.pdf]

IRMPD Spectroscopy of Homo- and Heterochiral Asparagine Proton-Bound Dimers in the Gas Phase

Åke Andersson,† Mathias Poline,‡ Kas J. Houthuijs,¶ Rianne E. van Outersterp,¶ Giel Berden,¶ Jos Oomens,¶ and Vitali Zhaunerchyk*,†

†*Department of Physics, University of Gothenburg, Gothenburg, Sweden*

‡*Department of Physics, Stockholm University, Stockholm, Sweden*

¶*Radboud University, Institute for Molecules and Materials, FELIX Laboratory, Toernooiveld 7, 6525ED, Nijmegen, The Netherlands*

E-mail: vitali.zhaunerchyk@physics.gu.se

1 Supporting Information for "IRMPD Spectroscopy of Homo- and Heterochiral Asparagine Proton-Bound Dimers in the Gas Phase"

1.1 Experimental spectra

Figure S1 – S3 show the experimental IRMPD spectra in greater detail. The intensities are the strongest in the range of 1000 – 1900 cm^{-1}, as indicated by the grid.

1.2 Calculated energies

Figure S4 shows calculated energies using different methods. For each the structure is optimized with B3LYP-GD3BJ, ωB97XD, or M06-2X using the 6-311++G** basis set. G4MP2 is then employed for a single-point calculation.

The results show that while electronic energy is not dependent on the choice of optimization method, Gibbs energy is. As an extreme example, the energy difference between DD-A1 and DD-B1 is -2.358, 3.258, or 6.157 $\mathrm{kJ\,mol^{-1}}$ when B3LYP-GD3BJ, ωB97XD, or M06-2X is used. For comparison, at room temperature $k_\mathrm{B}T = 2.494\,\mathrm{kJ\,mol^{-1}}$, which means that the relative abundances of conformers strongly depend on choice of method.

Table 1 lists the relative electronic energies E, Gibbs energies G, and abundances p of conformers.

1.3 Figures and Tables

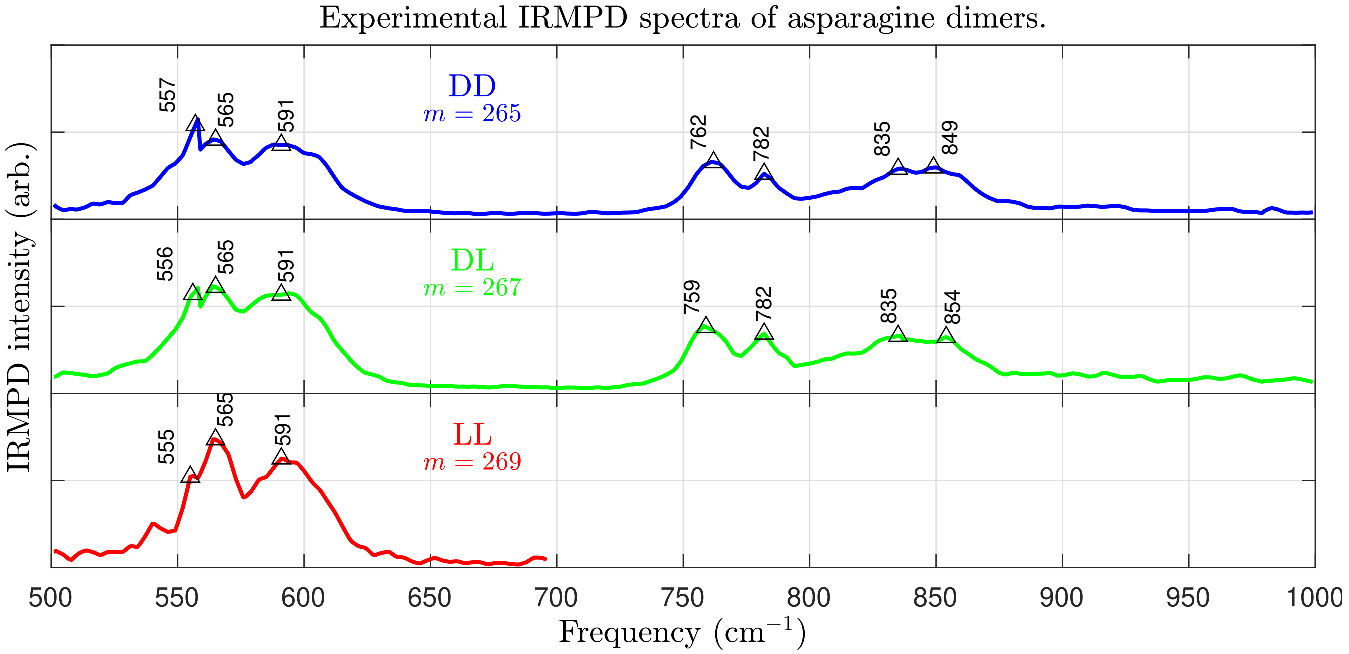

Figure S1: Experimental IRMPD spectra in the range of 500 – 1000 cm^{-1}.

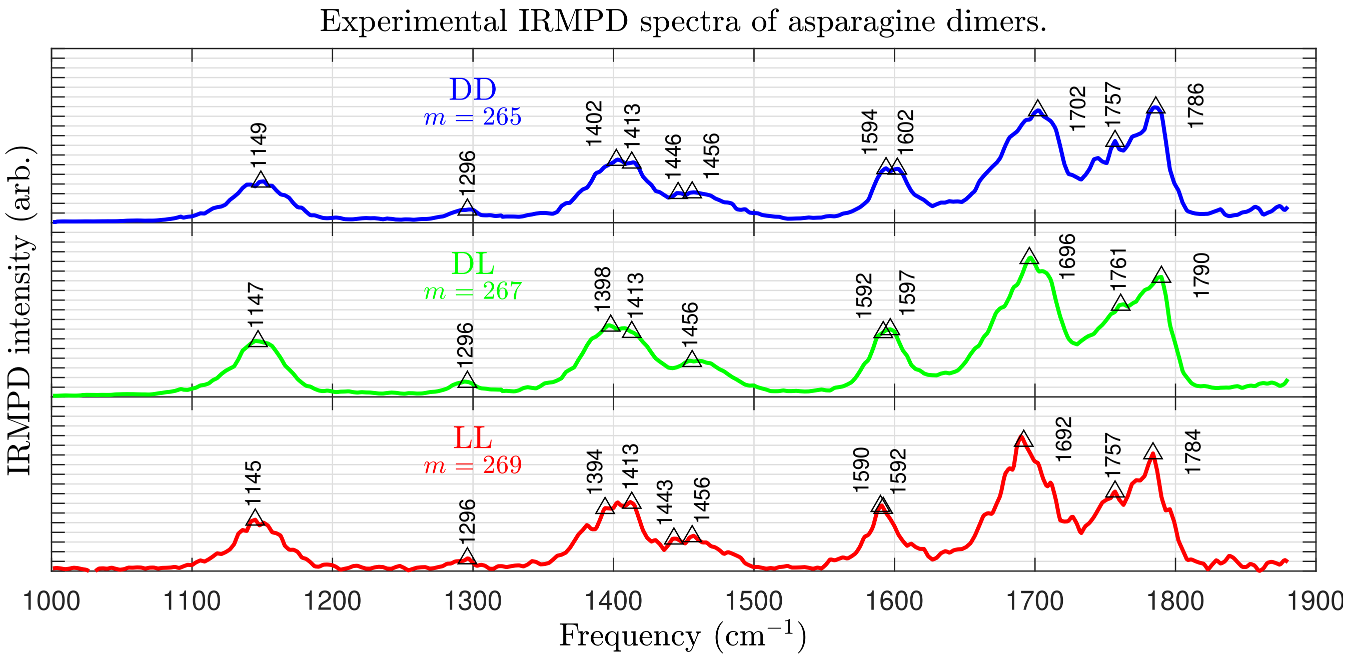

Figure S2: Experimental IRMPD spectra in the range of 1000 – 1900 cm^{-1}.

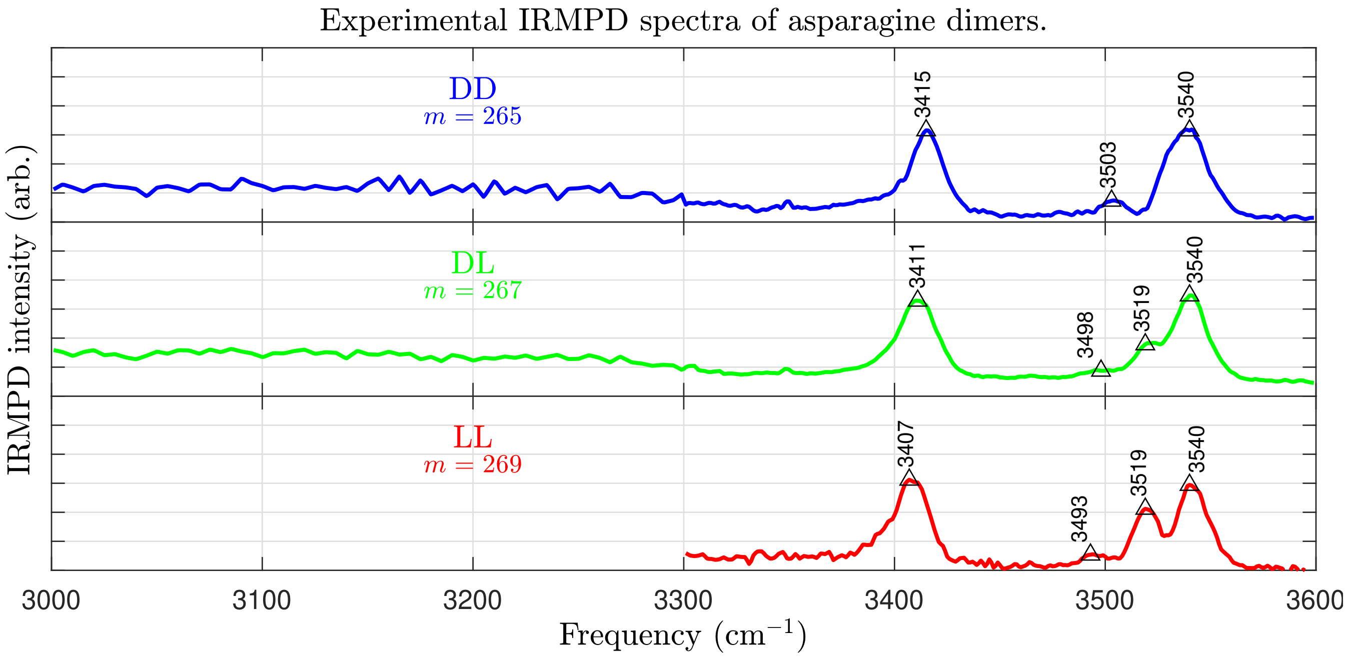

Figure S3: Experimental IRMPD spectra in the range of 3000 – 3600 cm^{-1}.

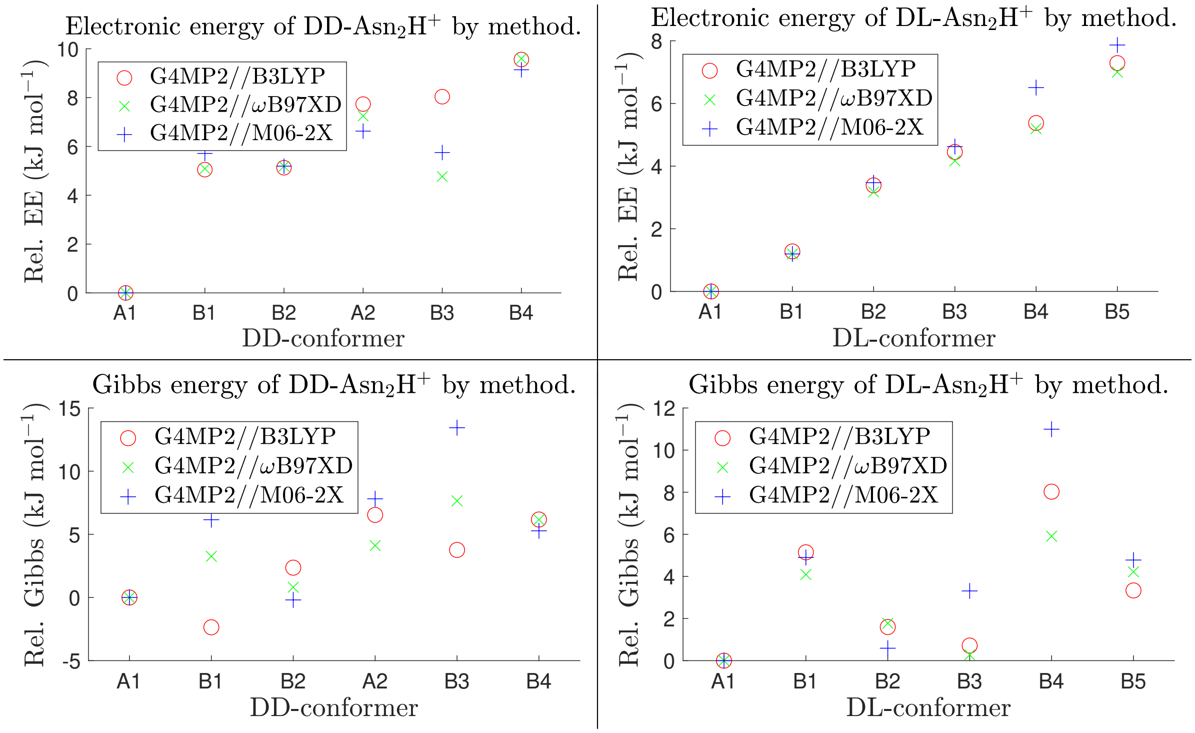

Figure S4: Computed relative (top) electronic and (bottom) Gibbs energies of the most stable (left) DD-Asn$_2$H$^+$ and (right) DL-Asn$_2$H$^+$ conformers using three different methods. The 3D-structure of each conformer is optimized with one of B3LYP-GD3BJ, ωB97XD, or M06-2X. G4MP2 is then employed for a single-point calculation. In all calculations the basis set is 6-311++G**.

Table 1: Relative electronic and Gibbs energies of the most stable conformers in $\mathrm{kJ\,mol^{-1}}$, calculated with B3LYP-GB3BJ/6-311++G. The first letter in dimer notation is the name of the amino acid where the proton is.**

Conf.	E	G_{300}	p_{300}	G_{100}	p_{100}
DD-A1	0.00	2.36	0.23	0.00	0.97
DD-B1	5.05	0.00	0.59	2.99	0.03
DD-B2	5.13	4.72	0.09	4.65	0.00
DD-A2	7.73	8.89	0.01	7.61	0.00
DD-B3	8.04	6.12	0.05	6.50	0.00
DD-B4	9.56	8.53	0.02	9.11	0.00
DL-A1	0.00	0.00	0.37	0.00	0.90
DL-B1	1.28	5.14	0.05	2.71	0.03
DL-B2	3.39	1.60	0.19	2.48	0.05
DL-B3	4.45	0.72	0.28	3.03	0.02
DL-B4	5.38	8.03	0.01	6.44	0.00
DL-B5	7.29	3.33	0.10	6.26	0.00
